# Diversion Alert: 1-Year Evaluation Across Northern New England, 2013–2014

**DOI:** 10.5888/pcd13.160229

**Published:** 2016-11-23

**Authors:** Sarah Levin Martin, Clare Desrosiers

**Affiliations:** 1 Husson University, School of Pharmacy, Bangor Maine; 2 Diversion Alert, Houlton, Maine.

## Abstract

This report describes Diversion Alert, a unique online tool aimed at reducing misuse and diversion of prescription drugs, and reports the results of a 1-year evaluation of Diversion Alert’s impact in Maine. We used a quasi-experimental research design to compare survey data in Maine with those of neighboring states (New Hampshire and Vermont, 2013 and 2014). Compared with their counterparts in New Hampshire and Vermont who did not use Diversion Alert, prescribers and pharmacists in Maine who used Diversion Alert increased their communication with patients and other providers involved in their patients’ care, became aware of patients arrested for prescription drugs possession or diversion, used best practices associated with prevention or detection of addiction and diversion more frequently, and attributed positive changes in their prescribing practices to Diversion Alert. In combination with other state and federal programs, Diversion Alert may be an effective tool to help prevent the misuse of opioid medications.

## Introduction

The epidemic of opioid overdose is well-documented (1), and it is known that prescription drug misuse and diversion are a part of the problem (2,3). The United States’ ongoing prescription drug abuse epidemic is leading to a dramatic increase in opioid overdoses and overdose deaths nationwide. Every day, 46 people die as a result of overdose from prescription painkillers (4). Including heroin overdoses makes deaths from opioid overdoses the leading cause of unintentional death for Americans, rising 14% from 2013 to 2014 (5). Although they are not the only source, physicians are the leading source of prescription painkillers for people at high risk for painkiller overdose (2). This statistic points to a fundamental concern associated with prescription abuse — a medical system that may unintentionally cause addiction rather than preventing and treating it. Medical professionals are ideally positioned to intervene with patients who struggle with addiction and could more effectively do so if given tools and resources that help them identify and respond to vulnerable patients (ie, those who are at risk for overdose or who are illegally diverting prescription medicines).

An array of interventions have been implemented nationally or at state levels to limit prescription opioid misuse (6). For example, naloxone availability and treatment programs have multiplied (7), changes have occurred in clinical guidelines and in the development of abuse resistant opioids (8), and adoption of prescription drug monitoring programs (PDMPs) has been almost universal (in 49 of 50 states), although characteristics of these programs vary by state (9). Although federal efforts, including grant funding, are substantial and span several federal agencies (eg, Substance Abuse and Mental Health Services Administration, Health Resources and Services Administration, Drug Enforcement Agency), there has not yet been a systematic approach to linking health system and law enforcement efforts as a means to more effectively address prescription drug abuse and diversion. Prescription drug diversion has been defined as the unlawful channeling of regulated pharmaceuticals from legal sources to the illicit marketplace (10). Diversion occurs at various points within the medical distribution system — via wholesale distributors, medical offices, retail pharmacies, or patients themselves (11).

It is well-recognized that the issue of drug abuse, misuse, and diversion must be approached from several angles. In this report, we outline the components of a potentially useful tool for linking medical and enforcement systems by providing drug arrest records to medical professionals. We also describe the preliminary results obtained to date on this innovative strategy.

## Diversion Alert

Diversion Alert (DA) is a program unique to Maine and was established in 2009 with federal funds from the Office of National Drug Control Policy’s Drug Free Communities Support Program, which were awarded to a community coalition in northern Maine. When federal funds expired in 2012, the program received state funding from the Maine Attorney General’s Office. Diversion Alert aims to 1) provide medical professionals with a source of information to identify patients who are at risk for overdose, in need of addiction treatment, or engaged in illegal pharmaceutical distribution; 2) increase attentiveness to prescribing practices; and 3) increase use of prescribing practices that help reduce abuse and diversion of prescription drugs. The core components of Diversion Alert are 1) monthly drug arrest reports from the previous month, consisting of public drug arrest data that are organized by geographic region (ie, by county and by state); 2) online, mobile-friendly, searchable drug arrest database containing an 11-month historical record of public drug arrest data submitted to Diversion Alert and accessible to registrants any time; and 3) research-based educational resources to assist in responding to patients charged with prescription drug or illegal drug crimes.

DA provides access to prescription (schedule II-IV) and illegal drug charge data for medical professionals (actively licensed pharmacists, prescribers, and medical office staff authorized to participate on behalf of prescribers or pharmacists), so they can respond to patients in need of intervention. Significantly, Diversion Alert distributes drug charge data rather than conviction data. There are 2 types of drug charges: an arrest and a summons. An arrest gives notice to a person that he or she is being charged with a crime. The person who is arrested is detained and kept in custody until he or she can post bail or the criminal charges are resolved. A summons gives notice to an individual that he or she is being charged with a crime, but the individual is not detained. In contrast, a conviction is a formal declaration by a court that a person has been found to have committed (is guilty of) a crime. The State must prove a person’s guilt beyond a reasonable doubt to obtain a conviction. The fact that a person has been arrested or received a summons does not guarantee that the person will be convicted of a crime.

Provision to prescribers of drug charge data, rather than conviction data, may be a point of concern; however, there are 3 keys points to consider. First, in Maine it takes from 12 to 18 months for a person charged with a drug crime to be convicted. Because individuals are generally released shortly after an arrest, there is a long period from charge to conviction during which a person could be putting himself or others at risk as a result of an untreated addiction or undetected diversion of pharmaceuticals. Therefore, it may be safer to use drug charge data as soon as they are available (within a few weeks of the date of charge) rather than to use conviction data. Second, anecdotal evidence from discussion with law enforcement in Maine suggests that more than 90% of drug charges end in a conviction. Additionally, a study conducted by the Bureau of Justice Statistics found that 93% of drug defendants adjudicated during 2006 were convicted (12). Most importantly, Diversion Alert is intended to be used as a resource to improve patient care and not as a punitive measure against patients. When a medical professional discovers a patient on a Diversion Alert report, that discovery is an indication both that something may be occurring and that additional work should be done (eg, talking to the patient and to other professionals who share the patient’s treatment, checking the prescription drug monitoring program) to get a more complete picture of what is going on with the patient.

As a public health intervention, Diversion Alert is based on “the public health model, which demonstrates that problems arise through relationships and interactions among an agent . . . a host . . . and the environment” (13). That is, in some instances prescription drug abuse and diversion arise from the interactions among the patient, the prescriber, and the prescribing environment within the medical system. In accordance with the Transtheoretical Model of behavior change (14), health care providers who begin using Diversion Alert will transition from being unaware of the extent of prescription drug abuse in Maine to a point at which they will be ready to change prescribing behaviors in response to patients they discover abusing or diverting prescriptions or to prevent future diversion and abuse. The specific prescribing behaviors the program seeks to increase are practices recommended in the literature (eg, urine drug screens, random pill counts, frequent use of prescription drug monitoring programs) for reducing abuse and diversion of prescription drugs for any person prescribed schedule II through IV controlled substances. By providing information to prescribers that aims to change prescriber perceptions and behaviors, patient addiction and diversion may be more effectively addressed and prevented, and fewer pharmaceuticals may be illegally diverted into communities.

## Methods

The Diversion Alert evaluation used a quasi-experimental, pre/post design with New Hampshire and Vermont as comparison groups. The Provider Awareness and Practice Survey, developed for this study and used as the basis for this evaluation, has 40 questions and exists both in paper form and online. The survey questions fall into the following categories: general (items 1–3 regarding professional role, state of practice, and zip code), beliefs about diversion (items 4–6), current practice with emphasis on communication and prescribing habits (items 7–19), use of universal precautions in practice (items 20–26), use of screenings and other assessments (items 27–35), and behavioral response to patients who have been arrested for illegal prescription drug activities (items 36–40). The survey was reviewed for face validity and content validity by 3 expert evaluators and medical and law enforcement professionals who serve on DA’s Statewide Advisory Board.

The Provider Awareness and Practice Survey, hosted on Survey Gizmo, was distributed during 2 periods, 1 year apart (summer/fall of 2013 and summer/fall of 2014) through bulk mailings to actively licensed prescribers and pharmacists in all 3 states. Mailing lists were obtained through professional associations, through databases maintained by states, or both. A cover letter with the Internet address and the paper form of the survey with a business-reply envelope were mailed to all prescribers and pharmacists in the states under study.

Descriptive statistics were used to calculate frequencies of responses. Analysis of Variance was used to detect difference across states, and *t* tests were used to detect differences within a state over time. Alpha was set at .05.

## Results

In 2013, 1,811 respondents participated in the pre-evaluation, and 782 respondents participated in the 2014 postevaluation (Table 1). Posttest respondents and their professional grouping indicated that most responders were prescribers, and 10% to 20% were pharmacists. Less than 9% identified as “medical office staff” or “other.”

**Table 1:** Provider Awareness and Practice Surveys Completed by State, Northern New England, United States, 2013–2014

State	No. of Completed Presurveys	No. of Completed Postsurveys (% of Prescribers)
Maine	862	202 (81.7)
New Hampshire	580	385 (76.1)
Vermont	369	195 (73.9)
**Total**	1,811	782

In 2013, 87% of Maine survey respondents reported yes to the item, “There is a prescription abuse/diversion problem in my local area.” One year later, 98% agreed. By 2014, nearly all respondents in Vermont believed that there was a prescription abuse/diversion problem in their local area (96%). In New Hampshire, 76% agreed. The percentages increased by more than 10% between 2013 and 2014 for Maine and Vermont but not for New Hampshire.

For the item, “In the past 6 months, I have become aware of patients in my care arrested for prescription drug possession or diversion,” 49% of Maine respondents said yes in 2014, significantly more than either New Hampshire (21%; *P* < .05) or Vermont (29.6%; *P* < .05).

With regard to communicating with health care providers who share a patient’s treatment plan, Maine respondents increased from 2013 to 2014 more than the other states (0.46 on a 4-point Likert scale [1 = never, 2 = somewhat, 3 = a lot, and 4 = all the time] compared with 0.04 for New Hampshire and 0.01 for Vermont). Communicating with law enforcement decreased from 2013 to 2014 for Maine respondents (−0.25 on 4-point Likert scale compared with −0.05 for Vermont and +0.04 for New Hampshire). By 2014, Maine respondents were more likely to have changed several of their prescribing practices than were respondents in New Hampshire or Vermont (Table 2).

**Table 2:** Prescribers’ Reported Behaviors Concerning Narcotics, Northern New England, United States, 2013–2014

Survey Item	Pretest Mean^a^			Posttest Mean^a^		
	Maine	New Hampshire	Vermont	Maine	New Hampshire	Vermont
I use patient agreements (or contracts) for patients who are prescribed ongoing therapy with narcotics.	3.06	2.8	2.81	3.31^b^	2.86	2.84^b^
I use patient agreements (or contracts) for patients who are prescribed ongoing therapy with controlled substances other than narcotics.	2.76	1.87	2.08	2.86^b^^,^^c^	2.08	2.16^b^
I use a screening tool to determine a patients' history of substance abuse or addiction.	2.4	1.89	2.08	2.39^c^	2.0	2.09
I order urine toxicology screens on new patients before prescribing a controlled substance.	2.63	1.76	2.06	2.26^c^	1.71	1.87
I order random urine toxicology screens on existing patients who are prescribed a controlled substance.	2.71	2.05	2.34	2.5	2.04	2.19
I speak to patients in my care about my knowledge of their prescription possession or diversion arrest.	2.82	2.33	2.49	2.71	2.34	2.52^b^
I share information with colleagues about how best to respond to patients I discover involved in illegal drug-related activities.	2.74	2.42	2.63	2.75	2.51^a^	2.57
I share information with colleagues about use of best practices for prescribing controlled substances.	2.7	2.29	2.45	2.64^c^	2.33^b^	2.37
I consult my state’s Prescription Drug Monitoring Program^d^	2.5	1.43	2.39	3.14^b^	1.56^b^	2.79^b^
If a patient in my care were arrested for a prescription drug-related crime, I would stop prescribing controlled substances to him/her.	3.38	3.65	3.61	3.51^b^^,^^e^^,^^f^	3.64	3.32
Stopped prescribing for patients who have been arrested	3.28	3.3	3.51	3.54^b^^,^^e^	3.68^b^	3.56^b^
Do not change meds for patients who have been arrested	2.79	2.65	1.38	1.32^b^^,^^e^	1.28^b^	1.27^b^
Refer for addiction counseling	NA			3.2^e^	3.19	3.16
Discharge patients who have been arrested	2.93	2.79	1.98	1.8^b^^,^^e^	2.12^b^	1.76^b^

a Values based on Likert scale scores, ranging from 1 = never to 4 = all the time.

b Indicates significant difference between pre- and post-test within each state.

c Significant difference between Maine and both Vermont and New Hampshire.

d New Hampshire did not have Prescription Drug Monitoring Program at the time of the surveys (established 2014).

e Significant difference between Maine and New Hampshire.

f Significant difference between Maine and Vermont.

In Maine only, at posttest, respondents were asked how they used DA. Eighty-four percent attributed improved attentiveness to prescribing to DA. More than half (59.3%) said they used it as a way to intervene with patients who were abusing or diverting prescriptions, and 40% used it as tool to screen new patients. Respondents were also asked to report the number of patients they discovered on a Diversion Alert report; 52% discovered at least 1 patient, with an average of 2.5 patients discovered per yes response.

## Discussion

It is noteworthy that Maine respondents increased their communications with others involved in the treatment of their patients but decreased their communications with law enforcement. This supports the idea that Diversion Alert is a tool for health care decision-making, not for law enforcement and legal action. Vermont also showed a significant decrease in communication with law enforcement whereas New Hampshire increased on this item. The absence of the PDMP program, and information provided by it, in New Hampshire may be associated with a higher degree of interaction with law enforcement as the basis for obtaining information about patients who may be involved in illegal activity. This point should be tested, because New Hampshire launched its PDMP in October of 2014.

In Maine, significant improvements were realized on communication and collaboration with patients through contracts, screening to garner additional information to guide prescribing and treatment decisions, and more conservative prescribing procedures to limit illegal use and diversion for patients who have been arrested. Moreover, Maine providers significantly decreased discharging patients who had been arrested, suggesting that they sought to provide needed health care to all patients while also attending to alternative prescribing for those who have been involved in illegal substance activity.

Piper and colleagues’ comprehensive analysis of data in Maine highlights the utility of Diversion Alert data to describe one aspect of the societal ill (15); opioids accounted for more than 50% of arrests between January 2014 and the first quarter of 2015 (11).

A 2014 survey of Maine pharmacists found that the most agreed-upon of several pressing issues identified were related to opioid misuse and diversion (16). Given this agreed-upon concern of pharmacists and their expanding role potentially afforded by the Affordable Care Act (17), Diversion Alert could be a useful tool, in addition to the PDMP, to screen patients before filling an opioid prescription. McCall and colleagues found in a study that linked Diversion Alert arrest data to patient PDMP records that Diversion Alert data complement PDMP data; 76% of individuals charged with prescription drug trafficking in 2014 did not have a matching record in Maine’s PDMP (18).

Our results are promising, but a limitation of our study is the inherent weakness in relying on survey data without psychometric properties established, a limitation that should be considered. In addition, the study design was unable to fully account for the historical threat to validity; nationally, attention is being given to the opioid epidemic, and alternative explanations such as statewide or national policy change are possible. However, more than 80% of survey respondents indicated improved attentiveness to prescribing as a result of Diversion Alert, and almost 60% reported using it as tool to intervene with patients; therefore, despite limitations, it appears likely that Diversion Alert could help prevent the abuse and misuse of opioid medications.
